# Chronic Pelvic Cellulitis and the Conditions Which Simulate It

**Published:** 1889-02

**Authors:** Virgil O. Hardon

**Affiliations:** Professor of Obstetrics and Diseases of Women and Children, Atlanta Medical College, Atlanta, Ga.


THE ATLANTA MEDICAL AND SURGICAL JOURNAL.

VOL. V. FEBRUARY, 1889. NO. 12.

### Original Communications.

### CHRONIC PELVIC CELLULITIS AND THE CONDITIONS WHICH SIMULATE IT.*

BY VIRGIL O. HARDON, M. D.,
Professor of Obstetrics and Diseases of Women and Children, Atlanta Medical College, Atlanta, Ga.

Chronic Pelvic Cellulitis is a subject which has attracted much attention during the last score of years, and in regard to which many diverse theories and opinions have been held by writers of authority. When Emmet called attention prominently to this disease and described it as the most frequent of all pathological conditions in the female pelvis, his views presented so much of plausibility that they were very generally accepted by the profession, and the treatment based thereon became well-nigh universal. The hot douche was adopted as the routine prescription with nearly every practitioner, and even the laity became cognizant of its reputed virtues. The fountain syringe became as in-

*Read before the Southern Surgical and Gynæcological Association, December 4, 1888.

dispensable an adjunct to every woman's toilet as the nail brush or the powder puff.

But after reaching this extreme the pendulum of professional opinion began to swing in the opposite direction, as might have been expected. A reaction from the views of Emmet resulted from the investigations of pathologists, and more especially from the observations of operators in the new field of abdominal surgery. Conditions which, when examined by the vagina, had been confidently diagnosticated as pelvic cellulitis, were found, when the abdomen was opened, either before or after death, to present phenomena entirely inconsistent with that diagnosis. From experiences of this kind a skepticism has arisen which is as extreme, and consequently as remote from the truth, as the earlier opinions of Emmet. The views of the extremists in this direction are well illustrated by a quotation from a private letter which I received from one of them. He writes: "I have seen and examined patients of professional friends who presented all the old familiar symptoms, both subjective and objective of cellulitis, peritonitis, or whatever you choose to call it, and have seen that abdomen opened within a few days or hours, and have *without a single exception** found diseased tubes and ovaries. * * * * Where I find what I used to treat as pelvic cellulitis, *I always* open the abdomen or throw up the case in some one else's favor who has more faith in its curability than I have."

As is usually the case, the truth lies between the two extremes of opinion. To assert that every case which presents hardness and tenderness in the vaginal roof, is a case of disease of the tubes or ovaries, is as great an error as to assert that such symptoms always indicate the existence of chronic pelvic cellulitis. The amazing progress of the science and art of gynæcology in the last few years has made it possible to discriminate between several conditions, which may give rise to the formation of hard masses in the pelvic cellular tissue, palpable to the touch. To describe these various conditions, their pathology and diagnosis, shall be the purpose of this paper.

In an article read nearly two years ago, I laid down the propo-

*Italics mine.

sition that "chronic pelvic cellulitis rarely, if ever, occurs except as a sequel of acute pelvic cellulitis."* Further study and observation have convinced me that I may make this statement still stronger, and assert that chronic pelvic cellulitis *never* occurs except as a sequel of acute pelvic cellulitis. It is gratifying to me to notice that my views on this subject are corroborated in the recently published work of Skene, one of the most clear-headed and practical observers in gynæcology that this country has ever produced. He says, "I think that the so-called chronic cellulitis, recognized and treated as such by some authorities, is nothing more than the products of the inflammation which remain after the inflammation itself has subsided."†

In order to elucidate my proposition it will be necessary to refer briefly to the pathology of acute pelvic cellulitis, in regard to which no material difference of opinion exists among the best authorities. Acute pelvic cellulitis in its clinical history presents three stages, a stage of effusion, a stage of solidification and a stage of absorption or of suppuration. The stage of effusion is short, lasting from twenty-four to forty-eight hours, and during this time serum from the blood is freely poured out through the walls of the blood-vessels into the meshes of the cellular tissue. During this stage the roof of the pelvis presents a bulging into the vagina and an œdematous or boggy feeling to the touch. The stage of solidification rapidly follows, and the effused serum solidifies into a firm mass, presenting to the examining finger a hard, resistant feeling, which has been aptly likened by Doherty to a board. By this solidification of effused serum, the womb is immovably fixed, and if the inflammation be limited to one side of the pelvis, the womb is pushed to the opposite side. The stage of resolution begins as soon as solidification is complete, and is characterized by the gradual absorption of the effused mass. This stage varies greatly in duration, according to the amount of exudation and the vigor and recuperative power of the patient. During this stage the intrinsic tendency is toward recovery, which may be hastened by appropriate treatment or

*Atlanta Medical and Surgical Journal, October 1887.

†Disease of Women, p. 559.

retarded by exposure or imprudence. In exceptional cases the inflammatory exudation breaks down into pus, forming a pelvic abscess, which, if not artificially evacuated, finds an exit by one of various channels, usually the vagina. Such an abscess may heal readily after the escape of its contents, or may continue to discharge for an indefinite length of time.

The question then arises, is it possible for a condition of exudation of plastic lymph into the pelvic cellular tissue to take place, independently of an acute inflammatory process? From the standpoint of pathology, this question must be answered in the negative. The process of acute inflammation alone, can lead to exudation of the plasma of the blood into the surrounding tissues. In the condition which is known as chronic inflammation, a term regarded by many pathologists as a misnomer, the phenomena are not those of exudation with solidification and subsequent absorption, but, instead of this, are found cell proliferation and hypertrophy of pre-existing tissues. No observer, as far as I am aware, has ever claimed to have found changes of this character in the pelvic cellular tissue.

In the class of cases in which Emmet claims that chronic pelvic cellulitis is most frequently found, without a history of previous attacks of acute pelvic cellulitis, the researches of Dr. Henry C. Coe, of New York, show that no changes in the tissues are found after death to correspond with the symptoms existing during life. In cases of laceration of the cervix, with thickening or hardness and tenderness in the broad ligaments, typical cases of chronic pelvic cellulitis from Emmet's point of view, *post mortem* examination revealed nothing to account for the hard masses which were palpable to the finger by vaginal examination during life. Coe says: "In by far the greater number of cases where a well-marked laceration of the cervix was present, there was absolutely no induration whatever in the broad ligaments. This thickening, increased tension, or whatever it is, which we find at the examining table, and promptly enter in our case books as cellulitis, frequently vanishes after death, when the natural tension of the tissues has disappeared. I would add briefly, that in only a single case have I felt satisfied that I found well-marked remains of a cellulitis in a case of laceration of the cervix. "*

The appearances thus described are in marked contrast with those disclosed by examination of the pelvic organs after an attack of acute pelvic cellulitis, when uterus, ovaries and tubes are found immovably embedded in a mass of plastic exudation, from which it is sometimes impossible to extricate them, except by literally digging them out. The condition in such cases is well described by Hervieux, as a result of *post mortem* examination of six cases, in which the disease had existed from four to twenty-two days. He says: " The broad ligament on the affected side is converted into a hard tissue of a blueish gray or violet color, creaking under the scalpel and presenting the appearance of cartilage. The two folds of peritoneum are indistinct as are also the connective tissue and aponeuroses, which separate them. There is only an indurated mass, in the midst of which the normal structures are confused and unrecognizable. The thickness of these indurations of the broad ligament varies from several millimetres to several centimetres. I have seen it reach the almost incredible thickness of four to five centimetres."†

Clinical observation furnishes evidence equally strong, and pointing in the same direction. If in a case of acute pelvic cellulitis, which has progressed through the stages of effusion and solidification, until it comes under the notice of the physician in the latter stage, a vaginal examination be made, the vaginal roof on one or both sides will be found to present a firm, resistant feeling to the finger, while the womb will be immovably fixed, so that any attempt to raise it will cause an increase of pain and, if force be used, may possibly be followed by a renewal of the acute inflammation. Under favorable treatment, the hard masses of plastic exudation will disappear in a period varying from one to four months. The subjective symptoms will be relieved only *pari passu* with the disappearance of the exudation in the cellular tissue. If the inflammatory process be limited to one side, the

---

*New York Medical Journal, May 15, 1886, p. 545.

†Archives de Tocologie, Nov. 15. 1887, p. 975.

womb will be pushed over to the opposite side, and can be restored to its normal axis in the pelvis only after absorption of the exudation.

In a case of so-called chronic pelvic cellulitis, in which there has been no history of a previous acute attack, if a similar examination be made, the womb will be found to be of greater than normal size, and low in the pelvis, so that the cervix is supported by the posterior vaginal wall. On one or both sides of the cervix, extending outward, are irregular hard masses which are tender to the touch. The womb is not fixed in its abnormally low position, but may be easily elevated or moved from side to side. When the womb is raised upon the finger so as to lift it from the posterior vaginal wall and restore it to its normal elevation, and is maintained in that position for ten or fifteen minutes, the hard masses on either side of the cervix will be felt to slowly melt away under the finger until they have nearly or quite disappeared. As soon as the womb be allowed to descend the masses will slowly return. If the womb be permanently kept at its normal elevation by the daily insertion of a tampon of cotton into the vagina, for one or two weeks, it will be found that the womb diminishes in size, the pain and other pelvic symptoms disappear, nervous symptoms are relieved, the patient gains flesh and strength, and once more begins to feel that life is worth living.

From the clinical as well as the pathological aspect of the two classes of cases, the conclusion is irresistible that no connection exists between them. In the one case we have a typical picture of the inflammatory process, while in the other not a single feature of that process is to be detected. What, then, is the significance of the hard masses which are felt on either side of the uterus, in the broad ligaments in so-called chronic pelvic cellulitis? The rapidity with which they disappear upon the elevation of the uterus in the pelvis, entirely negatives the theory that they are composed of any solid material, either exudation into the cellular tissue or hypertrophy of normal structures, while their immediate disappearance at death, as shown by the above quoted investigations of Coe, adds force to the negation. The only theory which appears to me to accord with these facts, is that the hard masses on either side of the cervix are accumulations of venous blood within the distented venus sinuses, which are so abundant and so large in this locality. Such accumulations can be caused only by some obstruction to the venous current at some point within the pelvis.

A study of the anatomical distribution of the veins of the pelvis and of the inevitable results of uterine congestion upon the pelvic circulation, lends confirmation to this theory. The uterine vein passes downward along the lateral border of the uterus, receiving at various points, horizontal branches from that organ. At a point about midway between the the external and internal os it passes outward through the cellular tissue to the wall of the bony pelvis and thence upward toward the heart. In its passage through the cellular tissue of the broad ligament, it loses the characteristics of a vein and develops into a series of enormous distensible sinuses. Throughout its course it is entirely free from valves. Any obstruction at any point in the uterine vein must, therefore, necessarily result in damming up the blood behind the obstruction and causing a distention of that vein.

Whenever an injury of any kind to the pelvic organs takes place during labor and remains unrepaired, involution is interfered with. The womb fails to return to its normal size and is, therefore, of greater than normal weight. Consequently the natural equilibrium between the uterine supports and the weight to be supported is destroyed, and the organ sinks below its normal level in the pelvis. Savage has shown that when the womb sinks below its normal level, traction is first exerted upon the cellular tissue of the broad ligaments, "especially where it surrounds and accompanies the uterine blood-vessels."* Traction upon an elastic tissue necessarily involves traction upon the blood-vessels within that tissue, and traction upon an elastic tube, like a blood-vessel, means diminution of its calibre and obstruction of the flow of blood through it. Hence, when the womb, in a state of sub-involution, from parturient injury, sinks below its level from increased weight, a condition exists which gives rise to obstruc-

*The Surgery, Surgical Pathology and Surgical Anatomy of the Female Pelvic Organs. New York, 1880, Plates XVIII and XIX.

tion of the uterine vein and its consequent distention. When the womb is raised the obstruction is removed, and the distention of the venous sinuses is at once relieved.

The beneficial effect of the vaginal douche of hot water in such cases admits of easy explanation. The action of hot water upon living tissue is to diminish the calibre of the blood vessels, and thus to lessen the amount of blood going to the part. The amount of blood to be returned through the pelvic veins, is, of course, proportionate to the amount of arterial blood received, and whatever lessens the one correspondingly lessens the other. Moreover, a diminution of the amount of blood going to the womb while that organ is in a state of sub-involution, permits a more rapid resumption of the normal process of retrograde metamorphosis, and consequently of a return of the womb to its normal weight, since, as stated by Thomas, "The element which sustains the disease is an excessive supply of blood."* In proportion as the weight of the womb is diminished, the organ rises towards its normal position in ths pelvis, and the causative factor in the production of venous stasis is removed. The recognition of the beneficial effects of the hot water treatment, as advocated by Emmet, does not therefore imply an acceptance of the theory upon which that treatment is based. The same results may be much more rapidly attained by keeping the womb supported at its normal level in the pelvis by a tampon of cotton in the vagina.

Of late, Emmet has to some extent receded from the strong position formerly held by him, and in a paper read at the eleventh meeting of the American Gynæcological Society he said, "In the past, when I have used the term 'thickening in the broad ligaments,' I have not supposed that there existed between the folds of the broad ligament any deposit of lymph as a product of local inflammation, for I have long known the fact that the blood-vessels are not, as a rule, destroyed by cellulitis, but in time, from long-continued obstruction to the circulation, and from a want afterward of the proper support which the connective tissue alone can give, the veins become greatly enlarged. I have already described elsewhere this condition of the blood-vessels, and when-

*Diseases of Women, New York, 1880, p. 317.

ever I have detected evidence of an old cellulitis with this so-termed 'thickening of the broad ligaments,' I have supposed that the enlargement was due to the dilated state of these veins."* It is difficult to reconcile Emmet's long recognition of these facts with his retention of the term chronic pelvic cellulitis, or with his remarks at the first meeting of the Alumni Association of the Woman's Hospital, in the course of which he said, "If there is thickening on one side of the vagina on a level with the cervix uteri, it must be cellulitis."†

There is another class of cases which were formerly regarded as chronic pelvic cellulitis, but whose true significance has been recognized only since the advent of abdominal surgery. I allude to disease of the Fallopian tubes and their distention by pus. Here we find masses on either side of the cervix, palpable through the vagina, and to a cursory examination closely simulating inflammatory deposits in the cellular tissue or distention of the pelvic venous sinuses. The womb in such cases is movable, and its elevation in the pelvis does not produce a disappearance of the enlargement. So closely may this condition simulate those to which I have already referred, that even so skillful a finger as Emmet's may not recognize the distinction. In the paper from which I have already quoted, he says, "During the past winter Professor Polk kindly invited me to witness an operation in his service at Bellevue Hospital for the removal of a diseased tube. The patient had suffered from a double laceration of the cervix uteri, and I had, at a previous examination, expressed the opinion that the subsequent condition was one of thickening and shortening of the left broad ligament from an old cellulitis. At the operation, to my surprise, no broad ligament was found, and the enlarged tube lay directly against the side of the vagina. The relative position, or line, with the fundus of the uterus and the ovary, remained unchanged, and yet the fingers of the operator, as they grasped the tube, could be distinctly felt from the vagina and in contact with its walls."

In an uncomplicated case of pyosalpinx, the diagnosis may be

* Gynæcological Transactions, Vol. XI.

† N. Y. Med. Journal, May 15, 1886, p 548.

readily made by attention to the distinctive features of the conditions with which it is most apt to be confounded. In the words of Bandl: "If with more or less severe inflammatory symptoms, masses form in the neighborhood of the cervix, or extend to the deeper portions of the pelvis, being doughy or soft at the beginning, but rapidly becoming harder, or if large, well defined swellings form in the true pelvis, in front of or behind the uterus, the process can be none other than phlegmonous inflammation of the cellular tissue."* On the other hand, the disappearance of the so-called thickening upon the elevation of the womb to its normal level, shows the case to be one of distention of the venous sinuses. But when, as is frequently the case, a pyosalpinx is complicated by acute pelvic cellulitis, or by venous engorgement, one condition may so mask the other, that a complete diagnosis will be difficult, or even impossible.

It often happens that acute pelvic cellulitis is excited by an extension of the inflammatory process from the tube to the neighboring cellular tissue. In such cases, one attack of cellulitis may follow another in rapid succession as long as the exciting cause exists, and the intervals between the attacks may be too short to allow of the absorption of the exudation. Yet this condition cannot properly be called chronic pelvic cellulitis, since each renewal of the inflammatory process is characterized by the three stages of effusion, solidification and absorption, which make up the clinical history of an isolated attack of acute pelvic cellulitis. The following quotation from Skene approximates very closely to my views on this subject: "Much has been said about chronic cellulitis, but I have never been able to recognize any such condition. Chronic suppuration in a badly drained abscess may go on for any length of time—this we often see; also, frequent or repeated attacks of cellulitis may occur. But a chronic or continuous inflammation, such as we see in inflammation of the mucous membranes, is something which I have never met with in practice. This is quite in accord with what we know of cellulitis elsewhere, where the process begins, progresses and ends, and recovery follows, or it may be that the inflammation progresses to the stage

*Cyclopedia of Obst. and Gyn., Vol. XII, p. 133.

of suppuration, and for some reason suppuration is kept up, but this is simply a chronic condition of one stage of the process."*

In a paper read before the Medical Association of Georgia in April, 1887,† I described a procedure by which I have been able to cut short attacks of acute pelvic cellulitis, when seen in the stage of effusion, before solidification has taken place. This procedure consists of drawing off by means of the aspirator the serum effused into the meshes of the cellular tissue, and thus effectually forestalling the later stages of solidification and absorption. In two cases of rapidly recurring attacks of pelvic cellulitis from disease of the tubes, I have pursued this course with the result of effectually jugulating such attacks, and thus preventing fresh deposits of plastic lymph. The following case will illustrate this mode of treatment:

Mrs. L. H. B., married, age 42, multipara, had suffered for many years from repeated attacks of pelvic inflammation, occurring at intervals of from three to nine months. These attacks had increased in intensity, until they were characterized by general peritonitis, delirium, extreme collapse and subsequent prostration, in addition to the usual symptoms of pelvic cellulitis. When first seen by me she was in the fourth day of such an attack and in appearance was almost moribund. She survived the attack, however, and in a fortnight was convalescent. Vaginal examination then revealed the pelvis completely blocked up by plastic exudation, the womb absolutely immovable. On the left side the inflammatory mass could be felt above the brim of the pelvis. By means of the hot vaginal douche, frequently repeated, and the application of Churchill's tincture of iodine to the vaginal roof and the abdomen, the absorption of this exudation was promoted so that in three months it had nearly all disappeared. The tumor projecting above the brim of the pelvis could then be felt by its characteristic outline to contain a distended tube. An operation for its removal was refused by the patient. About four months after the former attack I was suddenly summoned and found the patient suffering with high fever,

---

* Diseases of Women, p. 559.

† Atlanta Med and Surg Journal, August, 1887.

pain and tenderness in the abdomen, and frequent and painful micturition. She recognized the symptoms as those which always ushered in her attacks of pelvic inflammation. By vaginal examination I found the cellular tissue of both broad ligaments in a state of tumefaction and presenting to the touch a boggy, œdematous feeling, which I have learned to recognize as indicative of the stage of effusion of acute cellulitis. The patient was etherized and an respirator needle thrust into the pelvic cellular tissue at four successive points and between two and three ounces of serum withdrawn. The swelling of the tissues immediately subsided. When the patient recovered from the ether she expressed herself as relieved from the pelvic pain, the pulse and temperature rapidly fell and in twenty-four hours she was convalescent. Examination showed no deposit in the cellular tissue, although the distended tube could still be felt high up as before. It is somewhat remarkable that she has not had another attack of pelvic inflammation up to the present time, although thirteen months have now elapsed since the one referred to.

A method similar to this has been advocated by Hervot, under the name of "*ponction capillaire aspiratrice*,"* for the removal of encysted collections of serum in the progress of chronic pelvic cellulitis. From the reports of his cases I am led to believe that the condition treated by him was identical with the case just described. He failed, however, to recognize the occurrence of repeated attacks of acute pelvic cellulitis, and thus appears to have overlooked the principle upon which the success of his treatment was based.

It has been maintained by some authorities in gynæcology, that acute pelvic cellulitis does not occur as an idiopathic condition, but only by extension of inflammation from some neighboring organ, the uterus, the ovaries, or the tubes. W. Gill Wylie, for instance, says that "four-fifths of the so-called 'minor inflammations' are due to an extension of the inflammatory process through the tubes. The indurations which used to be called cellulitis are really such prolapsed tubes and ovaries surrounded by adhe-

*Archives de Tocologie, July 30, 1887.

sions."* Wm. M. Polk similarly says: "In one hundred cases of 'cellulitis,' the pathological conditions which we are taught belong to that disease were not found. Pelvic inflammation is the direct product of tubal disease."† My experience has led me to a different conclusion. While not denying the greater frequency of secondary cellulitis, I am sure that cases do occur in which the disease originates in the pelvic cellular tissue. Since I introduced the method of treatment referred to above, of aspiration of the tissues when seen in the stage of effusion, I have met with two cases in which the disease being effectually aborted by this means and the patient well in forty-eight hours, careful examination revealed no derangement of the uterus, ovaries, or tubes, and the subsequent history of the patient showed her to be free from pelvic disease. In each of these cases the patient was mulliparous and the cellulitis resulted from imprudence or exposure during menstruation, thus corroborating a recent statement by Goodell, that "in virgins the disease is due to exposure to cold at the catamenial period."‡

Yet another condition which simulates chronic pelvic cellulitis, and which was to me a source of error until its true nature was pointed out by Coe,** consists in a contraction of the utero-sacral ligaments, so that they stand out as firm cords extending backward from the cervix to the sacrum. Luschka has demonstrated the presence of muscular fibres in these ligaments, and has given to them the name of "*retractores uteri*."†† This condition conveys to the finger a sensation very similar to that of plastic exudation in the cellular tissue, and has been described by Emmet as one of the evidences of the existence of chronic pelvic cellulitis. I have, in several instances, been able to verify the observation of Coe, that when the patient is etherized, such indurations can no longer be felt, showing conclusively that they are not due to organic changes in the tissues. It is still further

---

* N. Y. Med. Journal, May 15, 1886, p. 548.

† American Journal of Obst., Oct. 1888, p.. 1039.

‡ American Journal of Obst., Oct. 1888, p. 1037.

** N Y. Med. Journal, May 15, 1886, p. 545.

†† Anatomie des Weiblichen Beckens, p. 361.

stated by Coe, that upon *post mortem* examination he has found that the condition no longer exists. An appreciation of the significance of these facts will doubtless prove a great source of relief to those who, like myself, have deemed it necessary, in accordance with the dictum of Emmet, to overcome this condition before undertaking any operation about the cervix. I can recall cases in which, as a preliminary to the operation of trachelorrhaphy, I have spent months in striving to overcome this utero-sacral contraction, thus entailing upon the patient an expenditure of time and money which my later experience has shown to be entirely unnecessary. I now disregard, as obstacles to operation, all conditions of hardness in the roof of the pelvis, except those in which the womb is immovably fixed by exudation in the broad ligaments. I am inclined to the opinion that even this condition might safely be disregarded, provided the pulse and temperature fail to show the presence of an actually existing acute inflammatory process.

